# How does a lifestyle intervention during pregnancy influence perceived barriers to leisure-time physical activity? The Norwegian fit for delivery study, a randomized controlled trial

**DOI:** 10.1186/s12884-018-1771-8

**Published:** 2018-05-03

**Authors:** Lene A. H. Haakstad, Ingvild Vistad, Linda Reme Sagedal, Hilde Lohne-Seiler, Monica K. Torstveit

**Affiliations:** 1 Department of Sports Medicine, Norwegian School of Sports Sciences, P.O Box 4014, Ullevål Stadion, 0806 Oslo, Norway; 2 Department of Obstetrics and Gynecology, Sørlandet Hospital, Kristiansand, Norway; 3 Faculty of Health and Sport Sciences, University of Agder, Kristiansand, Norway

**Keywords:** Barriers, Supervised exercise, Physical activity, Pregnancy, RCT

## Abstract

**Background:**

To develop effective health promotional and preventive prenatal programs, it is important to understand perceived barriers to leisure-time physical activity during pregnancy, including exercise and sport participation. The aims of the present study was 1) to assess the effect of prenatal lifestyle intervention on the perceived barrier to leisure-time physical activity during pregnancy and the first year after delivery and 2) identify the most important perceived barriers to leisure-time physical activity at multiple time points during and after pregnancy.

**Methods:**

This secondary analysis was part of the Norwegian Fit for Delivery study, a combined lifestyle intervention evaluated in a blinded, randomized controlled trial. Healthy, nulliparous women with singleton pregnancy of ≤20 gestational weeks, age ≥ 18 years and body mass index ≥19 kg/m^2^ were recruited via healthcare clinics in southern Norway, including urban and rural settings. Participants were randomized to either twice-weekly supervised exercise sessions and nutritional counselling (*n* = 303) or standard prenatal care (*n* = 303). The principal analysis was based on the participants who completed the standardized questionnaire assessing their perceived barriers to leisure-time physical activity at inclusion (gestational week 16, *n* = 589) and following intervention (gestational week 36, *n* = 509), as well as six months (*n* = 470) and 12 months (*n* = 424) postpartum.

**Results:**

Following intervention (gestation week 35.4 ± 1.0), a significant between-group difference in perceived barriers to leisure-time physical activity was found with respect to time constraints: *“... I do not have the time”* (intervention: 22 vs. control: 38, *p* = 0.030), mother-child safety concerns: *“... afraid to harm the baby”* (intervention: 8 vs. control: 25, *p* = 0.002) and self-efficacy: *“... I do not believe/think that I can do it”* (intervention: 3 vs. control: 10, *p* = 0.050). No positive effect was seen at postpartum follow-up. Intrapersonal factors (lack of time, energy and interest) were the most frequently perceived barriers, and consistent over time among all participants.

**Conclusion:**

The intervention had effect on intrapersonal perceived barriers in pregnancy, but not in the postpartum period. Perceived barriers to leisure-time physical activity were similar from early pregnancy to 12 months postpartum.

**Trial registration:**

ClinicalTrials.gov: NCT01001689, registered July 2, 2009.

## Background

Regular physical activity has favourable physiological and psychological health benefits for both the mother and the fetus [1]. Benefits include gestational weight gain control, enhanced cardiorespiratory fitness, attenuation of complaints including low back pain, pelvic girdle pain and urinary incontinence, prevention of gestational diabetes, gestational hypertension and preeclampsia, improved feeling of wellbeing, self-image and mood stability, as well as shorter labor in women who start labor spontaneously and decreased incidence of operative delivery [1–5].

Despite the well-established benefits of meeting recommendation of at least 150 min of moderate-intensity physical activity weekly [1]**,** studies have shown that the majority of pregnant women do not engage in regular maternal exercise, with trimester variations (13.4–24.8%) [6–9]. Therefore, more research and interventions aimed at maintaining or increasing pregnant women’s physical activity level are warranted.

There are several common types of perceived barriers to physical activity, including environmental, intrapersonal and interpersonal barriers. There is limited information regarding perceived barriers to leisure-time physical activity, including exercise and sport participation, in pregnant women and postnatal women [10]. There is, however, some evidence on the effect of lifestyle interventions in reducing barriers among the general adult and older population [11–13]. Pregnancy is characterised by several physical and emotional changes [1], and it is plausible that perceived barriers encountered pre-pregnancy, throughout pregnancy, and when entering motherhood are each unique and may differ [14, 15]. Pregnancy is also considered an ideal time for behaviour modification [1], and studies have shown that pregnant women may be particularly receptive to health messages [16, 17]. Hence, understanding how a prenatal lifestyle intervention affects perceived barriers is essential for the success of future trials and physical activity programmes. There is also a need for longitudinal documentation of perceived barriers to leisure-time physical activity after intervention is concluded. To our knowledge, no studies have investigated perceived barriers at different time-points during and after pregnancy, which may provide important insight about which factors are most influential across time. Hence, the aims of the present study were two-fold:

1. Assess the effect of prenatal lifestyle intervention on perceived barriers to leisure-time physical activity during pregnancy and the first year after delivery among Norwegian women
2. Identify which perceived barriers to leisure time physical activity that are most frequently reported across four time-points: time of inclusion (gestational week 16), at the end of the intervention (gestational week 36) and after the completion of the intervention; at six and 12 months postpartum.

## Methods

### Design and setting

This secondary analysis was part of the Norwegian Fit for Delivery study, a combined lifestyle intervention (supervised exercise sessions and nutritional counselling) evaluated in a blinded, randomized controlled trial. The trial was conducted in the prenatal health care system of southern Norway, comprising both urban and rural settings. The study protocol, including primary endpoints (maternal weight gain, newborn birth weight, glucose regulation, complications of pregnancy and delivery, and maternal weight retention up to 12 months postpartum) and elaboration of the randomization has been previously published [18].

### Participants and randomization

The Norwegian Fit for Delivery study included 606 nulliparous women recruited by midwives from eight healthcare clinics, between September 2009 and February 2013. The size of the trial was primarily based on power calculations for the assessment of prevalence of newborns with a birthweight > 4000 g, hypothesizing a reduction from 20% to 10%. In order to demonstrate a statistical difference, we calculated that we would need 198 women in each arm of the study. Further, we wanted to examine subgroups within our population, specifically women with BMI ≥25 and women who report low levels of physical activity at baseline measurements. We expect that our study will have a dropout rate of approximately 25%. Hence, to compensate for these factors, we recruited 303 women in each arm [18]. Inclusion criteria were ability to read, understand and speak Norwegian or English, singleton pregnancy within the first 20 weeks of gestation, age ≥ 18 years and a pre-pregnancy body mass index (BMI) ≥19 kg/m^2^. Exclusion criteria were pre-existing diabetes, physical disabilities that would preclude participation in the exercise program, on-going substance abuse, as well as planned relocation outside the study area before delivery.

**Fig. 1:**
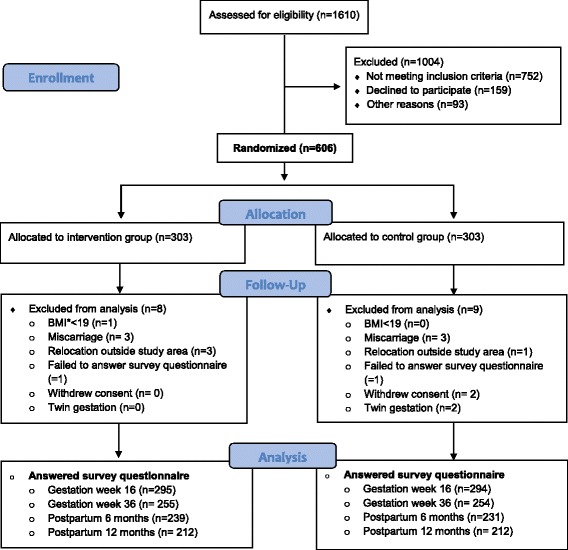
CONSORT 2010 Flow Diagram

Using a computer-generated list with 1:1 allocation ratio in blocks of 20, a research nurse, not involved in recruiting participants or carrying out the intervention, assigned participants consecutively to lifestyle intervention (*n* = 303) or control group (n = 303). A complete flow chart of the participants has been published previously [19]. Figure 1 shows a Consort diagram with respect to this secondary analysis.

### Intervention group

The dietary component of the trial consisted of two telephone consultations (with a doctor, clinical nutritionist or graduate student in public health) on dietary behaviour, invitation to a cooking class and one meeting that provided information on healthy diet and regular physical activity during pregnancy. Participants also received pamphlets and access to an Norwegian Fit for Delivery website, with recommendations for physical activity in pregnancy and dietary advice designed to increase awareness of food choices, decrease portion sizes and intake of snacks, and increase meal regularity and intake of water, fruits and vegetables [18]. Hillesund et al. (2016) have published on the dietary component of the trial [20].

From time of randomization until delivery, the intervention participants had access to twice-weekly supervised group sessions, offered at five different fitness centres, each lasting 60 min. The exercise program was tailored for pregnancy and followed contemporary guidelines [21], including 10 min of warm-up, 40 min combining cardiovascular exercise at moderate intensity and strength training, and finally10 minutes of stretching. Each session also included exercises for the pelvic floor muscles. Due to variations in maternal heart-rate responses to exercise [1], self-perceived exertion was set to 12–14 on the 6–20 Borg’s rating scale [22]. The fitness instructors (either physiotherapists or graduate students in sports science) were qualified to deliver antenatal sessions and registered attendance. Both participants and instructors were informed of indications to discontinue exercise, including vaginal bleeding and rupture of membranes [1].

Although practical and economic considerations limited classes to two per week, all women in the intervention group were encouraged to be physically active at moderate intensity on three additional days per week, lasting at least 30 min, in accordance with recommendations for physical activity during pregnancy [21].

### Control group

Participants in the control group received routine prenatal care in accordance with Norwegian standards, including eight routine prenatal contacts and one second-trimester ultrasound examination. Prenatal care is free of charge in Norway and provided through alternating visits with midwives and doctors.

There was no financial compensation to the participants, but all examinations and consultations, as well as exercise sessions were free of charge. In addition, two extra prenatal examinations, including ultrasound measurements, were provided in the third trimester for all trial participants.

### Outcome measure

A longitudinal approach was used to investigate the intervention- and the control groups perceived barriers to leisure-time physical activity across several time points, including exercise and sport participation.

#### Perceived barriers to leisure-time physical activity

The specific questionnaire section related to perceived barriers was presented in the same manner at all examination time-points, including 20 (during pregnancy) and 18 (postpartum) response-alternatives covering potential barriers that may limit regular leisure-time physical activity. Selection of more than one response was allowed, and the women were instructed to tick one or more perceived barriers that they found most important. The response options were based on reported barriers from previous studies in a pregnant population [23, 24] and a pilot testing completed among six research group members. The possible responses were *“insufficient time ( I do not have the time)”, “I cannot afford it”, “transportation problems”, “negative experiences”, “problems with mobility”, “I do not believe/think that I can do it”, “I do not have the energy”, “afraid to get hurt/injured”, “lack of interest (I would rather use my time on other things)”, “physical health/medical causes”, “lack of exercise companion (I do not have anyone to do physical activities with)”, “the scheduled time doesn’t fit me”, “lack of availability of exercise options (I do not know of anything available to me)”, “I am afraid to go out”,“ lack of activities that interest me”, “due to nausea”, “fear of urinary incontinence”, “pelvic girdle pain” and “afraid to harm the baby”.* Women could also tick *“other” t*o register perceived barriers not listed in the table, followed by a prompt to elaborate on this response using their own words. Average number of perceived barriers limiting regular physical activity in the intervention and control group was calculated by adding the number of different barriers that were ticked. For example, reporting *“... insufficient time”* and *“... I do not have the energy”*, added up as two barriers.

As recommended by Sallis et al. [25], as well as published in other research articles [24, 26], the perceived barriers were categorized into four main groups by socioecological framework: intrapersonal (health and not health related), interpersonal, neighbourhood or environment, and policy (Table 2).

Background information such as age, body weight, height, smoking habits, education, employment and household income were obtained from the questionnaire answered at inclusion. Physical activity level was assessed by the International Physical Activity Questionnaire shortform (IPAQ-SF). Participants completed the questionnaire either electronically (97.6%, Norwegian version only) or in print (2.4%, Norwegian or English version).

### Statistical analyses

All statistical analyses were conducted with SPSS Software V. 24 for Windows. Background variables and descriptive statistics are presented as mean (SD) or frequencies (n), unless otherwise specified. In total, 589, 509, 470 and 430 participants completed the standardized study questionnaire at time of inclusion (gestational week 16), at the end of the intervention (gestational week 36) and after the completion of the intervention; at six months and 12 months postpartum, respectively. There was no difference in background characteristics between the participants answering the questionnaire and those not responding. Hence, they were considered representative of the sample at inclusion. Chi-square analysis was performed to determine if there were group differences in distribution of various perceived barriers, categorized by socioecological framework, including sub-analyses of intervention participants adhering to ≥15 group-exercises (*n* = 141) with the control group. For expected cell values less than 5, Fisher’s exact test was used. Average number of perceived barriers limiting regular physical activity in the intervention and control group was tested using one-way repeated ANOVA. A *p*-value of ⩽0.05 was considered statistically significant.

## Results

### Study population and adherence

At trial inclusion, there were no statistically significant differences between the intervention- and control groups in background or health variables (Table 1), including self-reported moderate to vigorous physical activity (440 vs. 380 MET-minutes/week *p* = 0.523). According to pre-pregnancy BMI criteria, 21.2% and 7.4% were categorized as overweight (BMI 25–29.9 kg/m^2^) or obese (BMI ≥30 kg/m^2^) respectively. The majority were of Norwegian descent (92.1%) and only 23 (3.8%) participants were daily smokers, compared to approximately 8% in the general pregnant population in Norway (Public Health Report 2014).

**Table 1:** Baseline characteristics of participants (*n* = 589)

	Intervention group (*n* = 295)	Control group (*n* = 294)
Variable		
Age in years [Mean (SD^a^)]	27.9 (4.24)	28.1 (4.46)
Gestational week [Mean (SD)]	16.1 (2.47)	16.1 (2.44)
Pre-pregnancy BMI^b^, kg/m^2^ [Mean (SD)]	22.7 (21.0–25.7)	22.7 (20.9–25.0)
Meeting PA^c^ recommendations [*n* (%)]	72 (24.4)	65 (22.1)
Current smoker [*n* (%)]	8 (2.8)	15 (5.0)
Education [*n* (%)]		
< 4 years university or college	104 (35.4)	88 (29.9)
≥ 4 years university or college	96 (32.5)	113 (38.4)
Employment [*n* (%)]		
Outside home	240 (81.4)	256 (87.1)
Sick leave	6 (2.0)	5 (1.7)
Income total household (US dollar)^d^ [*n* (%)]		
Low (< 50,000 per year)	95 (32.2)	88 (29.9)
Moderate (50,000–87,500 per year)	82 (27.8)	81 (27.6)
Good (> 87,500 per year)	101 (34.2)	101 (34.4)
No response or missing [*n* (%)]	17 (5.8)	24 (8.1)

^a^SD; Standard Deviation

^b^BMI; Body Mass Index

^c^PA; Physical Activity, assessed with IPAQ-SF

^d^Classification are based on income and wealth statistics for Norwegian households in 2016

Among the intervention participants, 259 (87.5%) attended both dietary consultations, 28 (9.5%) one, and nine (3%) none. With respect to the physical activity part of the intervention, 274 (92.6%) attended at least one exercise session at their local fitness centre. Registered attendance varied between 1 and 38 sessions, with a mean of 15.5 ± 10.2.

### Effect of the intervention

Tables 2 and 3 show a summary of the perceived barriers to leisure-time physical activity grouped by socioecological framework, during pregnancy and postpartum reported in the intervention and control group. No statistically significant differences were found in socioecological barriers between the intervention and control participants prior to the intervention. After the intervention, there was a difference in three intrapersonal, not health related exercise barriers: *“... insufficient time (being too busy)*” (*p*=0.03)*, “... I do not believe that I will manage (low self-efficacy)”* (*p*= 0.050) and *“... fear of harm to the bab*y” (*p*=0.002). Six months postpartum, there was a trend towards higher prevalence of environmental barriers in the intervention group compared to the control group (*p*=0.06), with a significant difference in the proportion of women reporting *“... lack of activities that interest me”* (*p* = 0.005). At 12-months follow-up, we did not find any statistically significant differences between the two groups in types of identified perceived barriers.

**Table 2:** Perceived barriers to leisure-time physical activity during pregnancy, numbers at inclusion (n = 589) and at the end of the intervention (*n* = 509)

Barriers	Gestational week 16.1 ± 2.4			*P*-value	Gestational week 35.4 ± 1.0			*P*-value
	*n* (%)		% (95% CI^a^)		*n* (%)		% (95% CI^a^)	
	Intervention (*n* = 295)	Control (*n* = 294)	Group difference		Intervention (*n* = 255)	Control (*n* = 254)	Group difference	
Intrapersonal								
Health related								
... problems with mobility	5 (1.7)	5 (1.7)	0.0 (−2.4 to 2.4)	1.0	73 (24.7)	90 (30.6)	6.6 (−1.5 to 14.5)	0.119
... afraid to get hurt/injured	3 (1.0)	1 (0.3)	0.7 (−1.2 to 2.6)	0.624	17 (5.8)	13 (4.4)	1.5 (−2.7 to 5.9)	0.454
... physical health/medical causes	10 (3.4)	11 (3.7)	0.4 (−2.8 to 3.5)	0.824	33 (11.2)	36 (12.2)	1.1 (−4.9 to 7.1)	0.701
... due to nausea	2 (0.7)	1 (0.3)	0.3 (−1.2 to 2.1)	1.0	15 (5.1)	21 (7.1)	2.3 (−2.2 to 7.0)	0.302
... fear of urinary incontinence	0 (0.0)	1 (0.3)	0.3 (−0.9 to 1.8)	1.0	6 (2.0)	4 (1.4)	0.7 (−1.9 to 3.6)	0.752
...pelvic girdle pain	24 (8.1)	28 (9.5)	0,9 (−3.7 to 5.7)	0.562	90 (30.5)	93 (31.6)	1.0 (−7.3 to 9.3)	0.791
Not health related								
... insufficient time	119 (40.3)	122 (41.5)	0.8 (−7.0 to 8.7)	0.803	22 (8.6)	38 (14.9)	6.3 (0.1 to 12.1)	0.030
... negative experiences	15 (5.1)	12 (4.1)	1.0 (−2.5 to 4.5)	0.555	6 (2.0)	3 (1.0)	1.2 (−1.4 to 4.0)	0.505
... I do not believe I can do it	14 (4.7)	10 (3.4)	1.3 (−1.9 to 4.7)	0.405	3 (1.0)	10 (3.4)	2.7 (−0.1 to 6.0)	0.050
... I do not have the energy	102 (34.6)	102 (34.7)	0.1 (−7.5 to 7.8)	1.0	104 (35.3)	91 (31.0)	5.0 (−3.5 to 13.3)	0.258
...lack of interest	80 (27.1)	97 (33.0)	5.8 (−1.5 to 13.1)	0.129	17 (5.8)	27 (9.2)	3.9 (−1.1 to 8.9)	0.117
... I am afraid to go out	9 (3.1)	9 (3.1)	0.0 (−3.0 to 3.0)	1.0	3 (1.0)	1 (0.3)	0.7 (−1.2 to 3.0)	0.624
... afraid to harm to the baby	71 (24.1)	72 (24.5)	0.4 (−6.4 to 7.3)	0.924	8 (2.7)	25 (8.5)	6.7 (2.4 to 11.2)	0.002
Interpersonal								
... lack of exercise companion	57 (19.3)	41 (13.9)	5.4 (−0.5 to 11.5)	0.078	13 (4.4)	16 (5.4)	1.2 (−3.0 to 5.4)	0.568
Environmental								
... transportation problems	12 (4.1)	11 (3.7)	0.3 (−2.9 to 3.7)	0.832	9 (3.1)	3 (1.0)	2.4 (−0.4 to 5.5)	0.142
... the scheduled time doesn’t fit me	21 (7.1)	30 (10.2)	3.1 (−1.5 to 7.8)	0.188	6 (2.0)	4 (1.4)	0.8 (−1.9 to 3.6)	0.752
... lack of exercise options	2 (0.7)	4 (1.4)	0.7 (−1.3 to 2.8)	0.686	2 (0.7)	6 (2.0)	1.6 (−0.8 to 4.3)	0.286
... lack of activities that interests me	21 (7.1)	14 (4.8)	2.4 (−1.5 to 6.4)	0.223	7 (2.4)	7 (2.4)	0.0 (−3.4 to 3.2)	1.0
Policy ... I cannot afford it	28 (9.5)	27 (9.2)	0.3 (−4.4 to 5.2)	0.888	2 (0.7)	3 (1.0)	0.4 (−1.8 to 2.7)	1.0
Other reason	66 (22.4)	55 (18.7)	3.8 (−2.7 to 10.3)	0.264	54 (18.3)	52 (17.7)	0.9 (−6.2 to 7.9)	0.831

^a^CI; Confidence Interval

**Table 3:** Perceived barriers to leisure-time physical activity, numbers at six months (n = 470) and 12 months (n = 424) postpartum

Barriers	Postpartum 6.1 ± 0.8 months			*P*- value	Postpartum 12.1 ± 0.7 months			*P*-value
	*n* (%)		% (95% CI^a^)		*n* (%)		% (95% CI^a^)	
	Intervention (*n* = 213)	Control (*n* = 231)	Group difference		Intervention (*n* = 212)	Control (n = 212)	Group difference	
Intrapersonal								
Health related								
... problems with mobility	9 (3.8)	8 (3.5)	0.3 (−3.4 to 4.0)	1.0	3 (1.4)	5 (2.4)	0.9 (−2–0 to 4.1)	0.725
... afraid to get hurt/injured	1 (0.4)	1 (0.4)	0.0 (−1.9 to 2.0)	1.0	1 (0.5)	0	0.5 (−1.4 to 2.6)	1.0
... physical health/medical causes	7 (2.9)	9 (3.9)	0.9 (−2.5 to 4.6)	0.801	11 (5.2)	4 (1.9)	3.3 (−0.4 to 7.3)	0.114
... fear of urinary incontinence	6 (2.5)	4 (1.7)	0.9 (−2.0 to 4.1)	0.752	5 (0.5)	7 (3.3)	0.9 (−2.5 to 4.6)	0.772
...pelvic girdle pain	11 (4.6)	8 (3.4)	1.4 (−2.3 to 5.3)	0.642	11 (5.2)	9 (4.2)	0.9 (−3.3 to 5.3)	0.821
Not health related								
... insufficient time	107 (44.8)	97 (42.0)	5.7 (−3.2 to 14.4)	0.390	107 (50.5)	103 (48.6)	1.9 (−7.6 to 11.3)	0.733
... negative experiences	3 (1.3)	3 (1.3)	0.0 (−2.5 to 2.6)	1.0	4 (1.9)	2 (0.9)	0.9 (−1.8 to 2.9)	0.686
... I do not believe I can do it	3 (1.3)	8 (3.5)	2.2 (−0.7 to 5.5)	0.222	5 (0.5)	5 (2.4)	0.0 (−3.3 to 3.3)	1.0
... I do not have the energy	66 (27.6)	61 (26.4)	3.1 (−5.0 to 11.0)	0.618	60 (28.3)	59 (27.8)	0.5 (−8.1 to 9.0)	0.919
... lack of interest	53 (22.2)	67 (29.0)	6.8 (−1.1 to 14.6)	0.154	63 (29.7)	68 (32.1)	2.4 (−6.4 to 11.1)	0.622
... I am afraid to go out	1 (0.4)	2 (0.9)	0.4 (−1.6 to 2.7)	1.0	1 (0.5)	2 (0.9)	0.5 (−1.8 to 2.9)	1.0
Interpersonal								
... lack of exercise companion	19 (3.8)	20 (8.7)	0.7 (−4.4 to 5.9)	0.869	21 (9.9)	17 (8.0)	1.9 (−3.7 to 7.5)	0.503
Environmental								
... transportation problems	9 (3.8)	4 (1.7)	2.2 (−0.9 to 5.7)	0.262	3 (1.4)	4 (1.9)	0.5 (−2.4 to 3.5)	1.0
... the scheduled time doesn’t fit me	18 (7.5)	16 (6.9)	1.1 (−3.7 to 6.0)	0.724	21 (9.9)	16 (7.5)	2.4 (−3.1 to 7.9)	0.396
... lack of exercise options	2 (0.8)	1 (0.4)	0.5 (−1.6 to 2.7)	1.0	3 (1.4)	2 (0.9)	0.5 (−2.1 to 3.2)	1.0
... lack of activities that interests me	13 (5.4)	4 (1.7)	4.0 (0.5 to 7.8)	0.005	6 (2.8)	3 (1.4)	1.4 (−1.7 to 4.8)	0.505
Policy ... I cannot afford it	13 (5.4)	21 (9.1)	3.7 (−1.1 to 8.6)	0.158	16 (7.5)	16 (7.5)	0.0 (−5.2 to 5.2)	1.0
Other reason	41 (17.2)	43 (18.6)	1.5 (−5.5 to 8.4)	0.814	45 (21.2)	42 (19.8)	1.4 (−6.3 to 9.1)	0.728

^a^CI; Confidence Interval

Sub-analysis of participants with regular adherence to the prenatal exercise classes (attending ≥15 supervised sessions, *n* = 141) did not change the overall results of the Norwegian Fit for Delivery intervention trial on perceived barriers.

Both groups had a significant drop in total perceived barriers over time (Time 1: 2.15 ± 1.6, Time 2: 1.70 ± 1.5, Time 3: 1.25 ± 1.3 and Time 4: 1.24 ± 1.3, Wilks’Lambda = .964, *p* < 0.001, multivariate partial eta squared = .036), but with no statistically significant differences between the intervention and control group.

### Perceived barriers: Which are most influential across time?

At first measurement (gestational week 16.1 ± 2.4), the most frequently reported barriers to physical activity in the intervention and control group, respectively, were intrapersonal (not health) and related to *“... insufficient time”* (40.3% and 41.5%), *“... I do not have the energy”* (34.6% and 34.7%), and *“... lack of interest (I would rather use my time on other things)”* (27.1% and 33.0%).

In third trimester (gestational week 35.4 ± 1.0), health-related factors were perceived as the most important barriers in both the intervention (79.3%) and control group (87.4%), a significant increase compared to early pregnancy (15.5% vs. 83.4% for whole group, *p* < 001), with pelvic girdle pain and movement problems frequently mentioned. On the other hand, examining the whole group, less women cited *“...insufficient time”* (40.9% vs. 11.8%, *p* < 0.01) and *“... afraid to harm to the baby”* (24.3% vs. 5.6%, *p* < 0.001) compared to the first measurement, whereas *“... I do not have the energy”* was consistent throughout pregnancy.

At the follow-up measurements (postpartum months 6.1 ± 0.8 and 12.1 ± 0.7), the most commonly reported perceived barriers to leisure-time physical activity were intrapersonal, not health-related (Table 3), including the same perceived barriers as in gestation week 16 (insufficient time, no energy and interest). Few participants (less than 10%) mentioned interpersonal, environmental and policy barriers.

## Discussion

To our knowledge, no large-scale study has explored the effect of a lifestyle intervention on perceived barriers to leisure-time physical activity, or measured barriers across four time points from early gestation to 12-months postpartum. The main finding in this study was that the intervention group compared to the control group had fewer intrapersonal (not health related) barriers to leisure-time physical activity, including time constraints and mother-child safety concerns at late gestation. Low self-efficacy and lack of belief in one’s own ability to complete regular exercise was also reduced in the intervention group. Results from six- and 12-month follow-up showed that we did not succeed in altering perceived barriers beyond the period of active lifestyle modification. Counting only perceived barriers frequently cited (by more than 10% of the whole group), five major descriptive themes were identified. The most notable perceived barriers in early pregnancy and at both measurements postpartum belonged to the intrapersonal level: lack of time, no energy and no interest. Concern for the baby was also frequently reported at trial inclusion. At late gestation, health-related factors were perceived as the most important perceived barriers.

We included 606 healthy nulliparous women, and as shown in the Consort diagram (Fig. 1), there was loss to follow-up, with 589, 509, 470 and 424 participants completing the questionnaire at inclusion, at the end of the intervention and at six and 12 months postpartum. Hence, missing data might have reduced the statistical power of the study. We were not able to find any RCTs exploring the effect of a lifestyle intervention on perceived barriers to leisure-time physical activity in a pregnant population; hence, there were no comparable sample sizes.

As a result of individual randomisation, women living in close proximity and attending the same clinic were often in different trial groups. It is therefore possible that control participants were influenced to some extent, and that our analysis underestimates the effect of the intervention on perceived barriers, especially postpartum. Moreover, we can conjecture that we would have found a greater effect if the intervention components had been stronger, including implementation of behaviour change techniques such as goal setting and use of the Stages of change tool within the Transtheoretical Model [27, 28]. From a public health perspective, improving the new mother’s lifestyle habits may also positively influence the lifestyle of the new family [29].

Unfortunately, in our RCT, only 47% attended the minimum recommended number of exercise sessions. The reason for this lack of adherence is unclear; there is no data available regarding the reason for low participation. A fitness class of 60 min prescribed twice a week, combining cardiovascular exercise at moderate intensity and strength training, may be considered demanding. In addition, finding time to exercise is vital if an exercise program is to be followed. Exercise classes were at fixed time points, possibly excluding some participants for practical reasons. More flexible timetables for exercise classes and increased accessibility by public transport may increase adherence in future exercise interventions.

This study is unique in that we have repeated measurements of perceived barriers to leisure-time physical activity at four time-points, compared to most previous research that has identified barriers retrospectively. Prospective sampling is also more appropriate with respect to measuring different outcomes following an intervention [30].

The perceived barriers in the present study are consistent with a systematic review of barriers to exercise in pregnant populations [10], as well as previously published studies examining barriers to exercise among postnatal women [15, 31, 32]. The consistency of reported barriers from the beginning of pregnancy to 12 months postpartum may indicate that these challenges are difficult to overcome. Hence, future lifestyle interventions should be more precise in targeting the most persistent barriers, including teaching the participants strategies to increase the likelihood of success and increase exercise adherence. In the general population, initial face-to-face contact and telephone support may increase the adoption and maintenance of physical activity in middle-aged adults, particularly for those not interested in, or unable to attend, group exercise [11]. Qualitative literature has indicated the importance of appeal and enjoyment, as well as social aspects of interventions, to have an effect on perceived barriers [33].

In contrast to others [14, 26], we found a significant shift from not-health to health-related barriers from early to late pregnancy. It is also noteworthy that fear of harm to the baby was reported by nearly 25% at trial inclusion (mean gestational week 16), and was significantly reduced in both groups at the end of intervention (gestation week 36), with the largest drop in the intervention group (2.7% vs. 8.5%, *p* = 0.002). This may demonstrate that nulliparous women still view exercise as an unsafe activity and that they are uncertain about how exercise generally affects their fetus. However, the recent ACOG guidelines [1] states that no published studies have shown that, in the absence of medical complications, regular exercise during pregnancy will result in adverse effects on the fetus or increase the risk for early spontaneous abortion. Our results highlight the importance of precise and updated information on exercise and physical activity, based on the current guidelines, to be distributed to pregnant women.

We also found a significant effect on two other perceived barriers following the intervention: lack of time and low self-efficacy (*“...I do not believe/think that I can do it”)*. Hence, in health promotion, it may be essential to emphasize that a moderate amount of exercise may be achieved in a variety of ways, and that it is important to select activities that are enjoyable, fit into daily routines and do not require advanced skills. Walking can be recommended as a simple and easily available exercise mode for most pregnant women [23]. In addition, in view of the present results, there seems to be a need to establish exercise classes for pregnant women.

The influence of the lifestyle intervention on perceived barriers to leisure-time physical activity appeared to be achieved through lifestyle changes in pregnancy rather than postpartum, as perceived barriers for not performing leisure time physical activity for the intervention group approached those of the control group postpartum. Hence, it is likely that participants interpreted the intervention as a lifestyle to be adopted during pregnancy rather than maintained indefinitely. In addition, the lifestyle intervention, including supervised group exercises sessions, stopped after delivery, another plausible reason for no effect of the trial at six- and 12-months follow-up.

### Strengths and limitations

Unlike most studies, we did not recruit from only one maternity unit, but contacted women attending healthcare clinics in southern Norway, including urban and rural settings. Hence, a major strength is the pragmatic nature of the trial. Prospective randomised controlled design is also a major strength, and it is among the largest trials performed in a pregnant population studying a combined lifestyle-intervention including supervised exercise-classes following ACOG recommendations [21]. We identified perceived barriers to performing leisure time physical activity at four time-points, and there was little missing data throughout the study, counting all follow-up measurements: gestational week 36 (86%), six (78%) and 12-months (72%) postpartum. Furthermore, the questionnaire covered a broad range of perceived barriers, constructed upon other studies [23, 24], as well as categorized into four main groups by socioecological framework [25].

Despite the size of the trial, a limitation is that the sample size was not based on a- priori power calculation for perceived barriers to leisure-time physical activity. In addition, we did not investigate the validity and reliability of the questions asked to identify the most frequently reported barriers regarding exercise participation during and after pregnancy.

In the present study, emphasis was placed on including a relevant list of choices with respect to perceived barriers that would limit regular leisure-time physical activity during pregnancy and postpartum. Still, we might not have covered all perceived barriers specific to pregnancy (20 items) and following childbirth (18 items). The category “other barriers” had a high response rate at all measurements (about 20%), reflecting that we were not able to capture all relevant items. The participants were given the opportunity to elaborate in free-response section, but very few responded to this.

There are physiological and anatomical changes during pregnancy [34], and perceived barriers may be affected by common pregnancy complaints. At late gestation, health-related factors were the most frequently reported barriers. However, we included three pregnancy-specific symptoms (nausea, urinary incontinence and pelvic pain) only. Hence, it would have been advantageous if we had included a wider range of physical symptoms, as well as measured the impact of each barrier from “not relevant” (score 0) to “highly relevant” (score 10). We also recommend future lifestyle interventions to include measurements of other social cognitive components such as exercise motives/intentions [35, 36]. Where electronic communication is readily available, online surveys may be preferable. In the current study, nearly all participants answered the questionnaire electronically.

RCT’s are time consuming and involve cooperation from the participants. Pregnant women who volunteer for such a study of a lifestyle intervention may therefore be more interested and more attentive to these aspects than non-participants, creating a potential risk for selection bias. The pregnant women in this trial were all healthy and nulliparous, and predominantly white, European, of normal weight pre-pregnancy and with a high educational level, all of which may limit the external validity of our results.

## Conclusions

The Norwegian Fit for Delivery combined lifestyle intervention was successful in reducing intrapersonal barriers to leisure-time physical activity during pregnancy. However, this trial effect was not seen in the postpartum period. Lack of time, energy and interest were powerful perceived barriers among all participants and were consistent from early pregnancy to 12-months postpartum. Hence, future trials of interventions aiming to increase physical activity during pregnancy and into motherhood, should address perceived barriers at trial inclusion, as well as investigate how these perceived barriers may best be overcome, in order to positively affect exercise behaviour.

## Abbreviations

ACOG: American College of Obstetrics and Gynecology
BMI: Body Mass Index
CI: Confidence Interval
RCT: Randomized Controlled Trial
SD: Standard Deviation
